# Swimming attenuates tumor growth in CT-26 tumor-bearing mice and suppresses angiogenesis by mediating the HIF-1α/VEGFA pathway

**DOI:** 10.1515/biol-2022-0009

**Published:** 2022-02-28

**Authors:** Jiapeng Li, Liya Liu, Ying Cheng, Qiurong Xie, Meizhu Wu, Xiaoping Chen, Zuanfang Li, Haichun Chen, Jun Peng, Aling Shen

**Affiliations:** Department of Physical Education, Fujian University of Traditional Chinese Medicine, Fuzhou, Fujian 350122, China; Academy of Integrative Medicine and Fujian Key Laboratory of Integrative Medicine in Geriatrics, Fujian University of Traditional Chinese Medicine, 1 Qiuyang, Minhou Shangjie, Fuzhou, Fujian 350122, China; Provincial University Key Laboratory of Sport and Health Science, School of Physical Education and Sport Sciences, Fujian Normal University, Fuzhou, Fujian 350007, China

**Keywords:** swimming, colorectal cancer, angiogenesis, HIF-1α/VEGFA pathway

## Abstract

Low physical activity correlates with increased cancer risk in various cancer types, including colorectal cancer (CRC). However, the ways in which swimming can benefit CRC remain largely unknown. In this study, mice bearing tumors derived from CT-26 cells were randomly divided into the control and swimming groups. Mice in the swimming group were subjected to physical training (swimming) for 3 weeks. Compared with the control group, swimming clearly attenuated tumor volume and tumor weight in CT-26 tumor-bearing mice. RNA sequencing (RNA-seq) identified 715 upregulated and 629 downregulated transcripts (including VEGFA) in tumor tissues of mice in the swimming group. KEGG pathway analysis based on differentially expressed transcripts identified multiple enriched signaling pathways, including angiogenesis, hypoxia, and vascular endothelial growth factor (VEGF) pathways. Consistently, IHC analysis revealed that swimming significantly downregulated CD31, HIF-1α, VEGFA, and VEGFR2 protein expression in tumor tissues. In conclusion, swimming significantly attenuates tumor growth in CT-26 tumor-bearing mice by inhibiting tumor angiogenesis via the suppression of the HIF-1α/VEGFA pathway.

## 1 Introduction

Colorectal cancer (CRC) is one of the most common cancers worldwide, as more than 1.9 million new cases and 994,000 deaths were reported in 2020. Therefore, this disease ranks third in terms of incidence and second in mortality [1]. The development and progression of CRC are associated with multiple factors, including lifestyle, environmental factors, and genetic and epigenetic changes [2]. Despite the rapid development of various therapeutic strategies and combinations of these treatments, the outcomes of CRC patients remain unsatisfactory. Therefore, safer and less toxic therapeutic strategies are urgently needed.

Early evidence from observational studies has indicated that exercise exhibits a remarkable safety profile without obvious inherent toxicities and may even reduce the rate or severity of treatment-associated adverse events compared with other cancer therapeutics [3]. After the first preclinical evidence indicated that exercise inhibits tumor growth in a mouse model [4], a greater number of studies have demonstrated that exercise improves the quality of life in patients after surgery [5], improves chemotherapy efficacy [6], and is correlated with a reduced risk of recurrence and cancer-associated mortality [7]. Further investigation has indicated that exercise significantly attenuates tumor growth and metastasis, reduced serum levels of monocyte chemoattractant protein-1 (MCP-1), and decreased tumor hypoxia [8], and enhancement of intertumoral NK cell infiltration and activation [9] might be the essential underlying mechanisms. One study on CRC showed that increased physical activity after diagnosis was correlated with the reduction of cancer recurrence and mortality in stage III CRC patients [10] and reduction in the risk of CRC-specific and overall mortality in patients with stages I to III CRC [11]. However, the benefits and underlying mechanisms of exercise in patients with cancer, including CRC, require further exploration.

Multiple evidence suggested that low physical activity and high amounts of sedentary time correlate with increased cancer risk in various cancer types [12,13,14]. Previous systematic reviews and meta-analyses revealed that low physical activity and high amounts of sedentary time contribute to an increased risk of CRC [15,16]. By contrast, physical activity (including jogging and swimming) reduces CRC risk [17]. However, as a major aquatic exercise, the benefits of swimming and the underlying anti-CRC mechanisms remain largely unknown. Therefore, the current study was intended to assess the benefits of swimming with respect to tumor growth and to explore its underlying mechanisms against CRC.

## 2 Materials and methods

### 2.1 Materials

Fetal bovine serum (Cat. no. 1981614), RPMI-1640 (Cat. no. 1049101), 0.05% trypsin–EDTA (Cat. no. 293606), and penicillin-streptomycin were purchased from Thermo Fisher Scientific (Carlsbad, CA, USA). BD Matrigel (Cat. no. 354230) was obtained from BD Biosciences (San Jose, CA, USA). HIF-1α antibody (Cat. no. GTX127309) was provided by GeneTex Inc. (San Antonio, TX, USA). VEGFA (Cat no. ab1316), VEGFR2 (Cat no. ab2349), and CD31 (Cat no. ab28364) antibodies were purchased from Abcam (Cambridge Science Park, Cambridge, UK). Horseradish peroxidase (HRP)-conjugated secondary antibody (cat. no. Kit-0017) was obtained from Maixin Corp. (Fuzhou, Fujian, China). All other chemicals used were purchased from Solarbio Corp. (Beijing, China).

### 2.2 Cell culture

The CT-26 murine colon carcinoma cell line was purchased from the Shanghai Cell Bank of the Chinese Academy of Sciences (Shanghai, China). Cells were cultured in RPMI-1640 medium supplemented with 10% FBS and 1% penicillin (100 U/mL) and streptomycin (100 µg/mL) at 37°C and 5% of CO_2_ in a humidified incubator. Cells were subcultured at 80–90% confluency.

### 2.3 Animals

Twenty male BALB/c mice (age, 4–6 weeks; weight, 20 ± 2 g) were purchased from Shanghai SLAC Laboratory Animal Co., Ltd (Shanghai, China). Mice were housed under specific pathogen-free conditions at 22–26°C and 60 ± 5% humidity with a 12 h dark/light cycle. Food and water were provided *ad libitum* throughout the experiment.


**Ethical approval:** The research related to animal use has been complied with all the relevant national regulations and institutional policies for the care and use of animals and were approved by the Committee of Fujian University of Traditional Chinese Medicine (No. 2019-030).

### 2.4 Construction of the mouse xenograft model and measurement of tumor volume

After the mice were fed adaptively for one week, CT-26 cells (1 × 10^6^ cells/100 µL) in 100 µL of Matrigel (50%) were subcutaneously injected into the right flank area. The tumor volume was determined by measuring the major (*L*) and minor (*W*) diameters using an electronic vernier caliper and was calculated according to the following formula: tumor volume = *L* × *W* ^2^/2. To determine tumor growth, 20 tumor-bearing mice were randomly divided into the control group (*n* = 10) and the swimming group (*n* = 10) according to tumor volume three days after injection.

### 2.5 Exercise intervention program

In the pilot study, CT-26 tumor-bearing mice in the swimming group were subjected to physical training by swimming in water (30 ± 2°C) twice per day, 6 days per week. Initially, the mice typically swam voluntarily for approximately 10 min, after which they floated on the water and made intermittent swimming motions. The time was extended for 10 min each time until 30 min was reached. To induce mice to continuously swim for a longer duration, we used a stick to pull the water to drive them. During the experiment, the tumor volume and body weight were measured every three days. After 3 weeks, when the experiment ended, the mice were anesthetized with isoflurane and sacrificed. Tumor tissues were then removed and weighed.

### 2.6 RNA sequencing (RNA-Seq) and Kyoto Encyclopedia of Genes and Genomes (KEGG) analyses

Tumor tissues from each group (*n* = 6) were randomly selected, and total RNA was extracted with TRIzol (Tiangen, Beijing, China). RNA integrity was assessed using an Agilent 2100 Bioanalyzer (Agilent Technologies, Santa Clara, CA, USA), and RNA concentration was measured using a Qubit RNA Assay Kit in a Qubit Fluorometer (Invitrogen, CA, USA). A total of 1 µg RNA per sample was used. Briefly, the NEBNext rRNA Depletion Kit was used to remove rRNA from the total RNA sample. The NEBNext Ultra RNA Library Prep Kit for Illumina (NEB, Beijing, China) was used to construct the sequencing libraries according to the manufacturer’s instructions. Sequenced reads were trimmed for the adaptor sequence and then mapped to the hg38 whole genome using Hisat2 v2.0.5. Raw counts and fragments per kilobase million were calculated using StringTie v1.3.3. Differential expression analyses were performed using the limma package (cut-off > 2; *P* < 0.05). Differentially expressed transcripts (DETs) were identified using volcano plots and hierarchical clustering plots. KEGG pathway enrichment analysis was used to identify enriched signaling pathways represented among the DETs. The experiments were performed by CapitalBio (Beijing, China). The raw data were submitted to the Gene Expression Omnibus (GEO) (Submission No.: GSE 149405).

### 2.7 Immunohistochemistry

Immunohistochemistry was used to detect the expression of HIF-1α, VEGFA, VEGFR2, and CD31. Tumor tissues from each group were fixed in 4% paraformaldehyde (pH 7.4) for 24 h, processed, embedded in paraffin, and cut into 4 µm-thick sections. The slides containing tumor tissues were subjected to antigen retrieval and were then incubated with 3% hydrogen peroxide to block any endogenous peroxidase activity. After blocking nonspecific protein binding at 25°C for 10 min, the sections were incubated with primary antibodies against HIF-1α, VEGFA, VEGFR2, or CD31 (all diluted 1:200) at 4°C overnight. After the slides were washed in PBS, they were incubated with HRP-conjugated secondary antibody and then washed with PBS. The slides were then incubated with DAB chromogen, followed by counterstaining in diluted hematoxylin. After staining, images from each sample (five samples were randomly selected from each group) were obtained at ×400 magnification using a light microscope (LEICA: DM6000B, Wetzlar, Germany). Six fields of view were randomly selected for each slide, and the average percentage of positively stained cells in each field was counted using the true color multifunctional cell image analysis system Image-Pro Plus (Media Cybernetics, Rockville, MD, USA).

### 2.8 Statistical analysis

Data were presented as the mean ± SD for the indicated number of independently performed experiments. Statistical analysis was performed with Student’s *t*-test using SPSS24.0. Differences with *P* < 0.05 were considered statistically significant.

### 2.9 Data availability

The RNA-seq data associated with this article are available in the GEO repository [GEO Submission: GSE 149405].

## 3 Results

### 3.1 Swimming attenuates growth of CT-26 cell-derived tumors *in vivo*

**Figure 1:**
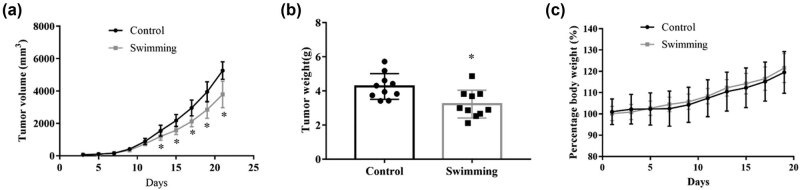
Effects of swimming on the growth of tumors derived from transplanted colorectal cancer cells in mice. (a) Tumor volume was monitored during the exercise period for 21 days. (b) Tumor weight was determined using an electronic scale at the end of the experiment. (c) The body weights of the mice were recorded during the experiment. Data are presented as the mean ± SD. * *P* < 0.05, vs control.

To assess the benefits of exercise on tumor growth, CT-26 cells were transplanted into BALB/c mice, followed by quantitative daily swimming. Tumor volume was monitored and clearly indicated that it was decreased in the swimming group compared with the control group (Figure 1a; *P* < 0.05). Consistently, a significant decrease in the tumor weight was observed in the swimming group compared to that in the control group (Figure 1b; *P* < 0.05). Moreover, monitoring of body weights did not reveal any obvious differences between the control and swimming groups (Figure 1c; *P* > 0.05). These data indicate that swimming significantly attenuates the growth of CT-26 cell-derived tumors *in vivo*.

### 3.2 Swimming regulates the expression of multiple genes in CT-26 cell-derived tumors

**Figure 2:**
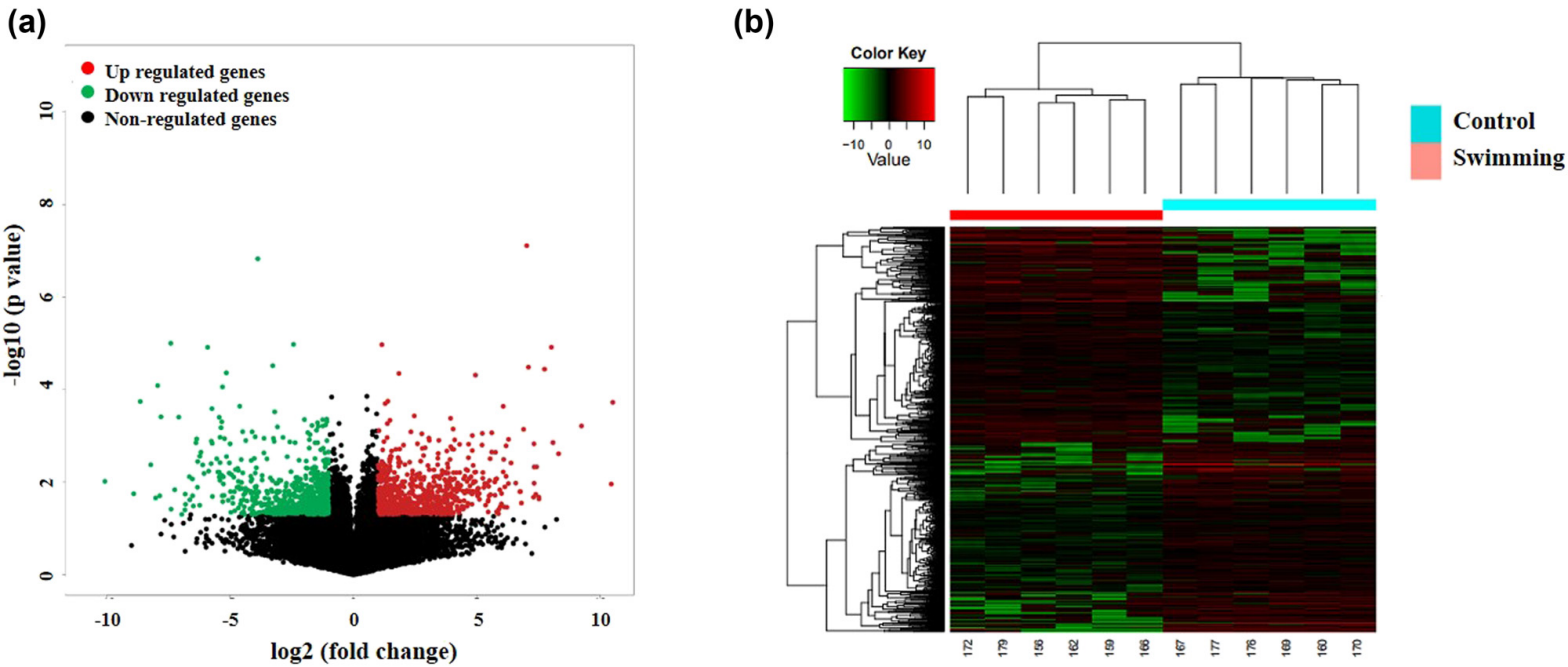
Identification of DETs. RNA-Seq was performed to determine the DETs in tumor tissues between the control and swimming groups. The DETs are presented using volcano plots (a) and hierarchical clustering plots (b). Cut-off >2, *P* < 0.05.

To further explore the underlying mechanism by which swimming attenuates tumor growth, RNA-seq was performed to DETs in tumor tissues between the control and swimming groups. As shown in Figure 2a and b (GEO Submission: GSE149405), we found 715 upregulated and 629 downregulated transcripts in the swimming group compared with the control group. The expression level of multiple genes associated with tumor growth, such as STAT3, PDGFA, PLD2, and PIK3R2, showed a significant decrease (Table S1). Table 1 also shows the top 20 altered DETs. These data suggest that swimming attenuates tumor growth by targeting multiple genes.

**Table 1:** Top 20 of different genes

ID	log FC	*P* value	Symbol
ENSMUST00000168776	9.24465	0.000611	2-Sep
ENSMUST00000105265	8.316306	0.002433	Cnot2
ENSMUST00000107846	7.745867	3.62 × 10^−5^	Clta
ENSMUST00000089776	7.327142	0.004677	Cep152
ENSMUST00000087582	7.324338726	0.010539031	Hnrnpm
ENSMUST00000119398	7.302732096	0.021052217	Fgfr1
ENSMUST00000025841	7.094498	3.27 × 10^−5^	Mus81
ENSMUST00000145960	7.025383	7.73 × 10^−8^	Ipo8
ENSMUST00000169734	6.768333	0.015967	Vps53
ENSMUST00000117179	6.744676	0.016023	Fgfr1
ENSMUST00000178282	6.387904	0.003841	Igha
ENSMUST00000150759	6.286162	0.001185	Unk
ENSMUST00000203193	6.280975	0.01071	8-Mar
ENSMUST00000212205	6.187989	0.007475	Wwp2
ENSMUST00000107847	6.002754	0.017555	Clta
ENSMUST00000106513	5.998047	0.029482	Mknk1
ENSMUST00000098080	5.950979	0.033919	Dcun1d3
ENSMUST00000124408	5.805759	0.008427	Asph
ENSMUST00000084027	5.802834	0.043785	Fgfr1
ENSMUST00000172638	5.757515	0.00226	Prdm5
ENSMUST00000187609	−8.64438	0.00018	Nupr1
ENSMUST00000173154	−7.94081	8.17 × 10^−5^	Exosc10
ENSMUST00000154428	−7.80568	0.000387	Unc45a
ENSMUST00000206592	−7.40941	9.95 × 10^−6^	Stambp
ENSMUST00000079896	−7.08613	0.000393	Tmem192
ENSMUST00000217929	−6.8355	0.039698	Epb41l2
ENSMUST00000155905	−6.82367	0.018103	Tex10
ENSMUST00000175778	−6.81097	0.030131	Sbf1
ENSMUST00000098826	−6.35686	0.001161	Dlc1
ENSMUST00000201575	−6.31666	0.003582	Ctbp1
ENSMUST00000107209	−6.23803	0.031545	Gabpb2
ENSMUST00000196204	−6.23232	0.002753	Gbp4
ENSMUST00000105964	−6.18801	0.002259	Gmeb1
ENSMUST00000160134	−6.06014	0.006475	Dab2
ENSMUST00000212378	−5.91456	1.21 × 10^−5^	Rpl18a
ENSMUST00000132520	−5.76178	0.001297	Nadsyn1
ENSMUST00000020681	−5.49563	0.006015	Slu7
ENSMUST00000226740	−5.44789	0.006064	Gnl3
ENSMUST00000156314	−5.40884	0.001071	Rnf20
ENSMUST00000216055	−5.371	0.000664	Gm48362

### 3.3 Swimming suppresses angiogenesis in CT-26 cell-derived tumors

**Figure 3:**
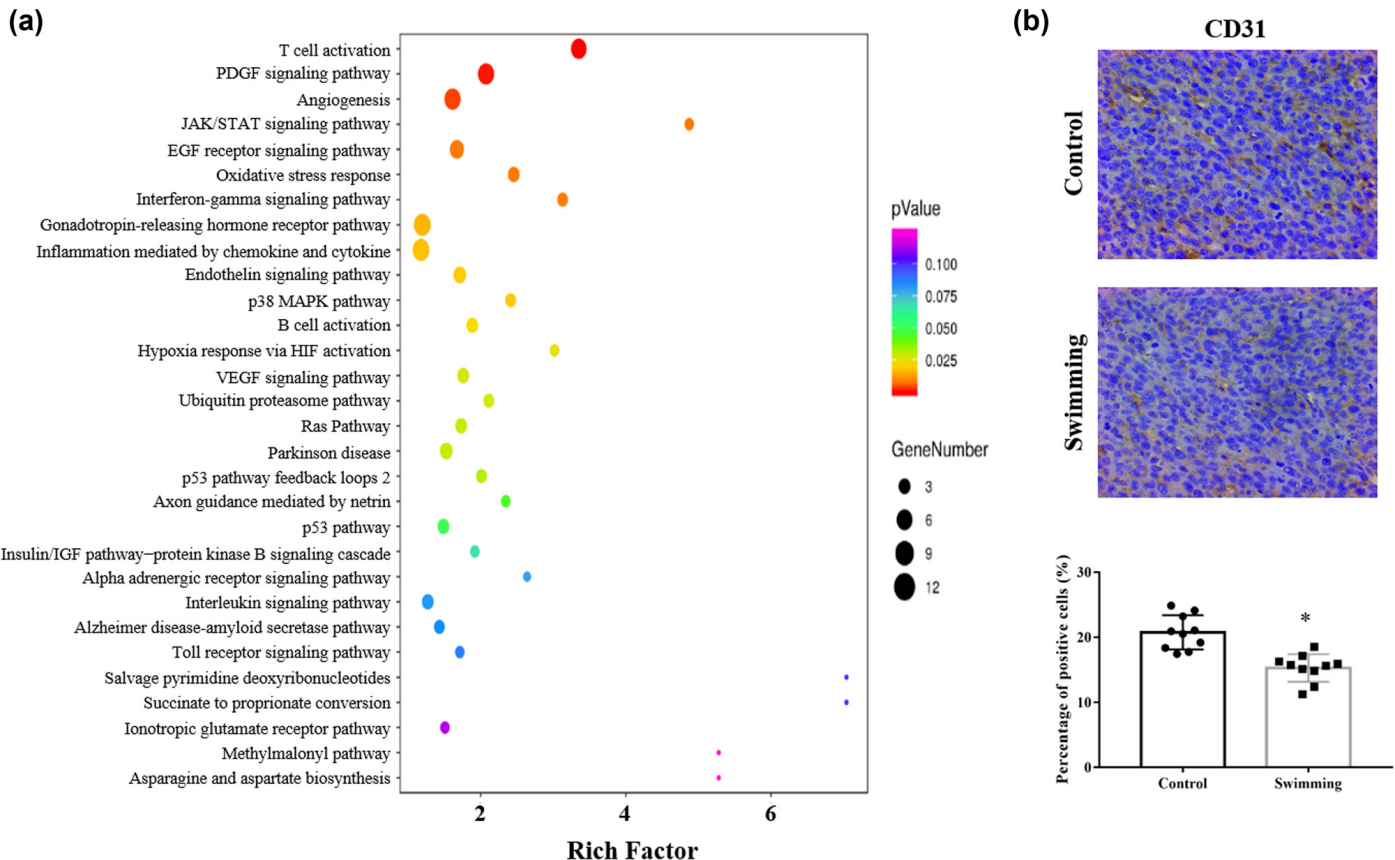
Effects of swimming on related signaling pathways and tumor angiogenesis. (a) KEGG pathway enrichment analysis based on DETs was used to identify related pathways. (b) IHC analysis was performed to determine CD31 expression in tumor tissues from mice in both the control and swimming groups; representative images were obtained at ×400 magnification. The average percentage of positively stained cells was counted using Image-Pro Plus. Data are presented as the mean ± SD. *P < 0.05, vs control.

To further analyze the involved signaling pathway, enrichment analysis of pathways based on DETs was performed. As shown in Figure 3a, multiple signaling pathways were enriched, including the angiogenesis, hypoxia, and VEGF pathways. To verify the mechanism by which swimming regulates tumor angiogenesis, IHC was performed to detect the expression of CD31. Compared with the control group, swimming significantly reduced CD31 expression in tumor tissues (Figure 3b), which demonstrates the attenuation of tumor angiogenesis due to swimming.

### 3.4 Swimming inhibits the HIF-1α/VEGFA signaling pathway

**Figure 4:**
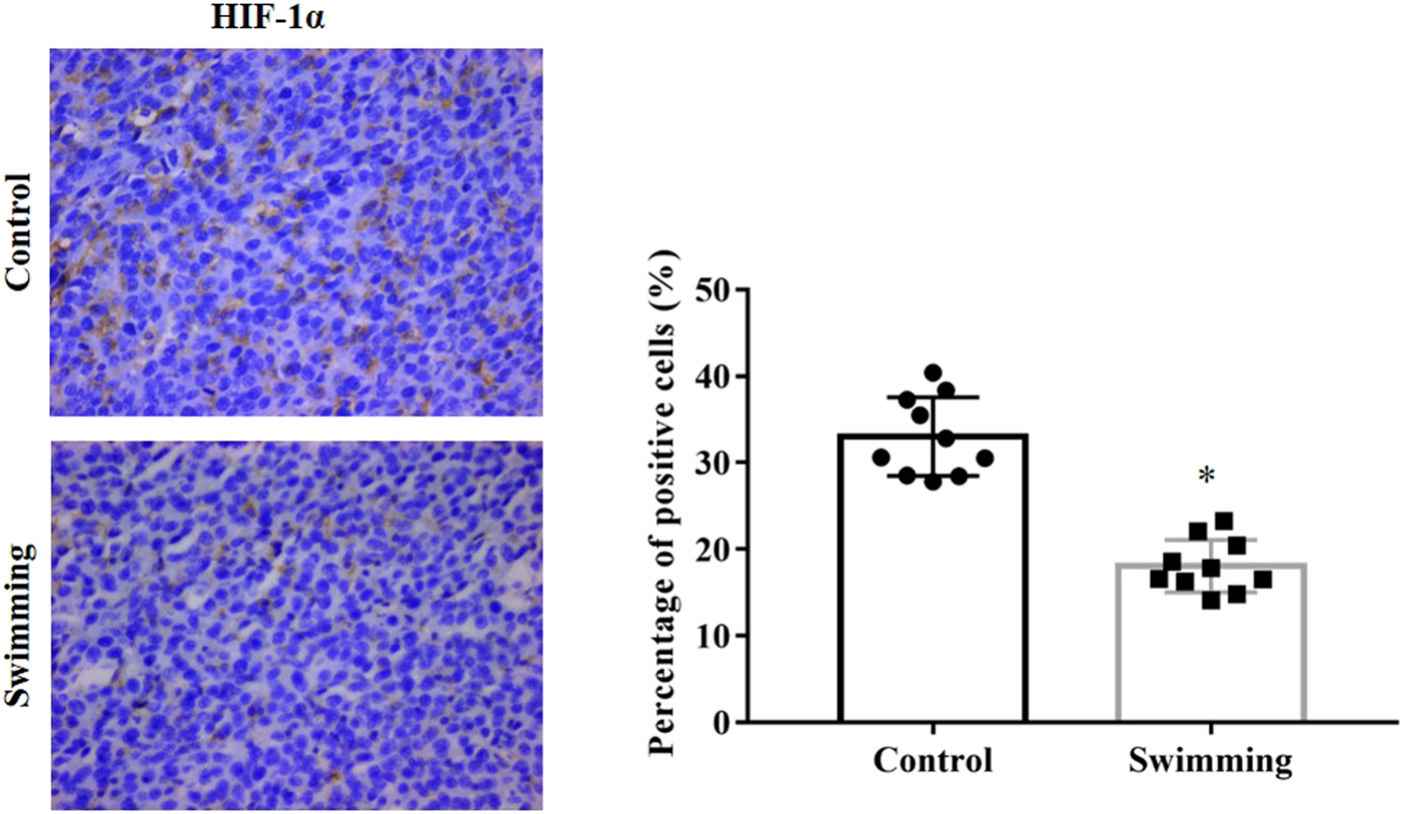
Effect of swimming on HIF-1α expression in tumor tissues. IHC analysis was performed to determine HIF-1α expression in tumor tissues from mice in both the control and swimming groups; representative images were obtained at ×400 magnification. The average percentage of positively stained cells was counted using Image-Pro Plus. Data are presented as the mean ± SD. *P < 0.05, vs control.

**Figure 5:**
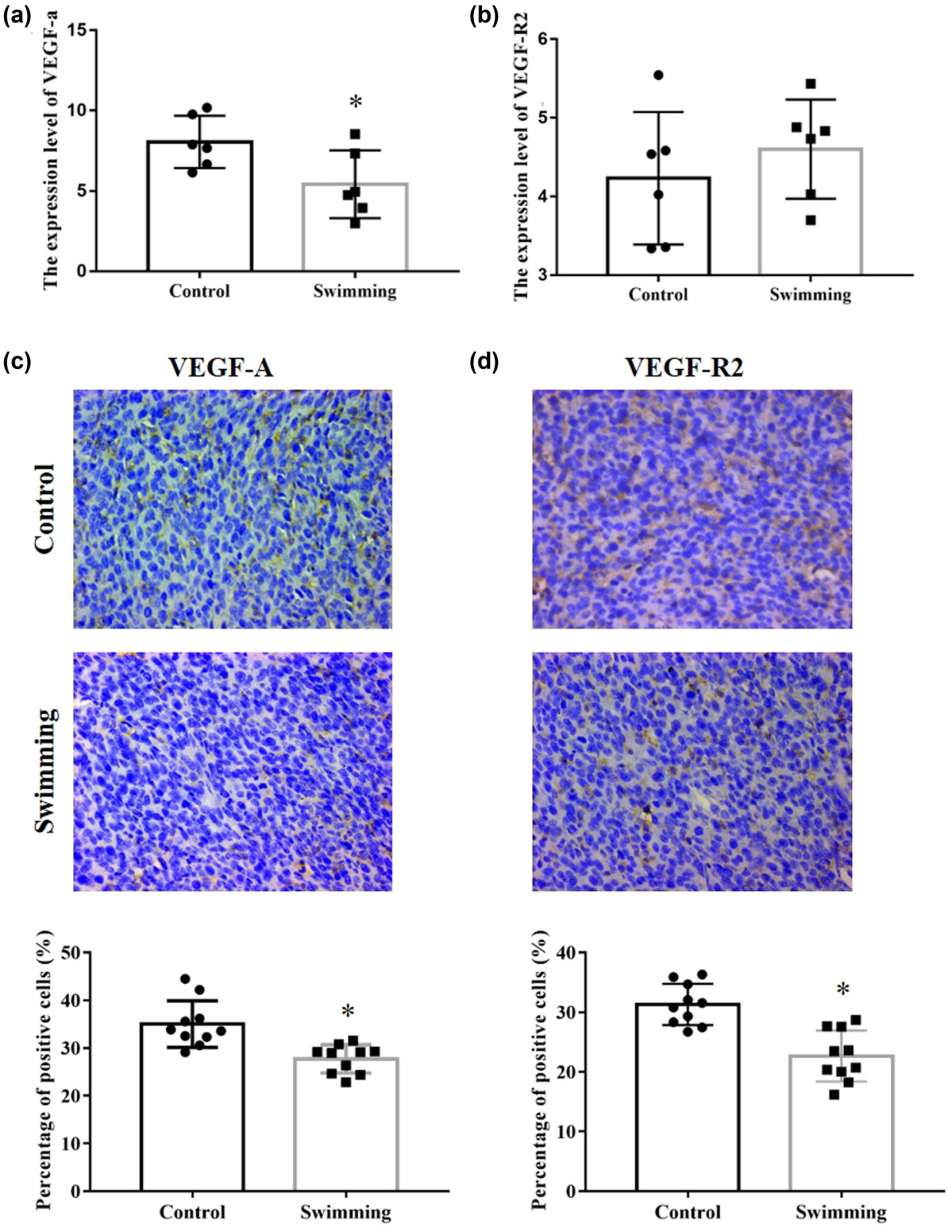
Effects of swimming on VEGFA and VEGFR2 expression in tumor tissues. The mRNA expression level of VEGFA (a) and VEGFR2 (b) in tumor tissues between control and swimming groups was analyzed by the RNA-Seq result. IHC analysis was performed to determine the expression of VEGFA (c) and VEGFR2 (d) in tumor tissues of mice in both the control and swimming groups; representative images were obtained at ×400 magnification. The average percentage of positively stained cells was counted using Image-Pro Plus. Data are presented as the mean ± SD. *P < 0.05, vs control.

Owing to the essential role of HIF-1α in tumor angiogenesis, we further detected the expression of HIF-1α. As shown in Figure 4, HIF-1α expression at the protein level was significantly decreased in the swimming group compared with the control group (*P* < 0.05). Furthermore, as essential downstream effectors of the HIF-1α pathway, VEGFA and VEGFR2 expressions were analyzed from RNA-Seq results and confirmed by IHC analysis. As shown in Figure 5a and b, the mRNA expression of VEGFA was significantly downregulated in tumor tissues of the swimming group (*P* < 0.05; vs control group), while the mRNA of VEGFR2 remained unchanged in tumor tissues between the control and swimming groups (*P* > 0.05). Moreover, expression of both VEGFA and VEGFR2 proteins was obviously decreased in tumor tissues of the swimming group (*P* < 0.05; vs control group). These results suggest that the suppression of the HIF-1α/VEGFA/VEGFR2 signaling pathway might be an important underlying mechanism by which swimming attenuates tumor growth *in vivo*.

## 4 Discussion

Increasing evidence has revealed that exercise results in multiple benefits, including suppression of tumor growth [4], improvements in quality of life [5], increased chemotherapy efficacy [6], and reductions in the risk of recurrence and cancer-associated mortality [7]. However, as a major form of aquatic exercise, the benefits of swimming in CRC remain largely unknown. In the current study, we demonstrated that swimming clearly attenuated the growth of CT-26 cell-derived tumors *in vivo*. Mechanistic studies identified 715 upregulated and 629 downregulated transcripts (including VEGFA) in tumor tissues derived from CT-26 cells *in vivo* after swimming. Further pathway analysis revealed significant enrichment of multiple pathways, including angiogenesis, hypoxia, and VEGF signaling pathways. Consistently, swimming also reduced the protein expression of CD31, HIF-1α, VEGFA, and VEGFR2, which suggests that the essential role of swimming is the suppression of tumor angiogenesis and that the inhibition of the HIF-1α/VEGFA/VEGFR2 axis might be an underlying mechanism by which swimming attenuates CRC tumor growth.

Previous studies in mice demonstrated that 30–60 min/day of swimming leads to a protective effect against cancer in mice [22,23]. Consistently, our current study revealed that swimming significantly alleviated tumor growth in CT-26 tumor-bearing mice. These studies indicated the benefit of swimming on tumors. However, owing to the contrary effects of the exercise of varying intensities in different diseases and states, including cancer [24] and inflammation [25], the benefit of different intensities of swimming on tumor growth should be further explored. Moreover, the effects of swimming on the quality of life and the underlying mechanism should be further investigated.

Although it has been reported that exercise protects against the development of certain cancers and lowers the risk of recurrence, the underlying mechanism by which swimming achieves this is largely unknown. To explore the complicated mechanism of swimming on CRC, identification of DETs using RNA-seq technology revealed that swimming led to 715 upregulated and 629 downregulated transcripts. Among these DETs, multiple genes, including STAT3 [18], PDGFA [19], PLD2 [20], and PIK3R2 [21], have been reported to be involved in tumor growth. Moreover, swimming altered the expression of some genes with less involvement or no known involvement in cancer, which should be further explored in future studies. To further investigate the involved signaling pathway, KEGG pathway enrichment analysis was used to identify the enriched signaling pathways based on DETs. Multiple signaling pathways, including the angiogenesis, hypoxia, and VEGF pathways were significantly enriched, which encouraged us to further explore the underlying mechanism by which swimming attenuates tumor angiogenesis and the HIF-1α/VEGFA pathway activation.

As a central mechanism for tumor growth, angiogenesis plays an essential role in tumor development and growth of human CRC [26,27]. Therefore, targeting tumor angiogenesis represents a novel strategy to combat CRC [28]. Previous studies revealed that exercise promotes vascular maturity [28], increases blood flow [29], and reduces the number of blood vessels [30,31]. Consistently, our current study demonstrated that swimming obviously downregulated CD31 expression, which suggests reduced microvessel density (MVD) and angiogenesis. Hypoxia is a common characteristic of solid tumors and plays an essential role in promoting tumor angiogenesis [32]. Under hypoxic conditions, the key mediator HIF-1α accumulates, dimerizes with HIF-1β, translocates into the nucleus, and binds to cis-acting hypoxia-response elements in target genes leading to increased transcription [33]. In our current study, RNA-Seq did not find the change of HIF-1A mRNA expression, while the determination of HIF-1α protein expression indicated that swimming clearly attenuated HIF-1α expression, which suggests the improvement effect of swimming on hypoxic conditions, consistent with previous studies [6,34]. These studies suggest that swimming might attenuate the activation of HIF-1α by increasing the protein expression of HIF-1α but did not affect its mRNA expression. However, the translocation of HIF-1α should be further explored in a future study.

Moreover, as a transcription factor, accumulated HIF-1α mediates the expression of more than 200 target genes, including VEGFA [35,36], which promotes endothelial cell proliferation and angiogenesis by binding to its receptor VEGFR2 [37]. As expected, swimming obviously reduced both the mRNA and protein expression of VEGFA and protein expression of VEGFR2, which might be one of the underlying mechanisms by which swimming suppresses tumor angiogenesis. However, the regulatory effects of swimming on hypoxia and angiogenesis should be further addressed, especially the distinction among different exercise intensities, times, and environments. Additionally, enriched signaling pathways other than HIF-1α/VEGFA should be investigated in tumor tissues after swimming.

In conclusion, swimming significantly attenuates the growth of CT-26 cell-derived tumors *in vivo*, reduces tumor angiogenesis, and downregulates the expression of HIF-1α, VEGFA, and its receptor VEGFR2. These studies suggest that swimming is a safe and viable intervention strategy for cancer patients. However, the effect of swimming on tumor vascular normalization, perfusion, oxygen transport, and anaerobic glucose metabolism, as well as its underlying mechanisms, should be further explored.
